# Jinzhi and fecal microbiota transplantation: a comparative review of historical and modern microbial therapeutics

**DOI:** 10.3389/fmicb.2026.1700764

**Published:** 2026-03-17

**Authors:** Muqi Li, Lijiao Dai, Yang Yang, Siyu Chen, Jing Ma, Peimin Feng

**Affiliations:** 1 Department of Gastroenterology, Hospital of Chengdu University of Traditional Chinese Medicine, Chengdu, Sichuan, China; 2 Department of Endocrinology, Hospital of Chengdu University of Traditional Chinese Medicine, Chengdu, Sichuan, China

**Keywords:** administration, donor, fecal microbiota transplantation, flora characteristics, FMT, Jinzhi, loess, microbial composition

## Abstract

Since its formal introduction in 1958, fecal microbiota transplantation (FMT) has gained prominence. However, challenges remain in standardizing protocols and optimizing efficacy. This review provides a systematic comparison between the historical practice of Jinzhi and modern FMT, focusing on their preparation methodologies. We hypothesize that specific, underexplored features of Jinzhi preparation could inform and refine current FMT practices. Specifically, we propose that the utilization of adolescent donors, underground low-temperature fermentation, and the careful consideration of seasonal timing, all integral to Jinzhi’s traditional protocol, may offer novel insights and testable hypotheses for enhancing microbial diversity, functionality, and therapeutic stability in FMT. By bridging this ancient wisdom with modern microbiome science, we aim to outline a novel and actionable framework for developing the next generation of microbiota-based therapeutics, urging future research to empirically test these historically inspired hypotheses.

## 1 Introduction

The gut microbiota constitutes the most extensive and sophisticated microbial ecosystem within the human organism. This microecological system performs crucial functions, including metabolic regulation, immunomodulation, nutrient absorption, pathogen resistance, and neuroregulation, which are essential for maintaining physiological homeostasis (Adak and Khan, 2019). Dysbiosis of this microbial ecosystem is strongly linked to various diseases, including inflammatory bowel disease (IBD), metabolic syndrome (MetS), malignancies, among others (Grochowska et al., 2019; Jiao et al., 2020; Liu et al., 2021; Nesci et al., 2023; Qiu et al., 2022; Scheithauer et al., 2020). Gut microbiota modulation has emerged as a cornerstone therapeutic strategy for various pathological conditions. Current clinical interventions utilizing probiotic and prebiotic supplementation show limited efficacy in addressing severe microbial dysbiosis (Yadav et al., 2022).

FMT is a clinical procedure that transfers processed donor microbiota into the gastrointestinal tract and has demonstrated rapid restoration of compromised gut microbiota, with clinically validated efficacy in managing refractory dysbiosis-related conditions, particularly recurrent *Clostridioides difficile* infection (rCDI) (Rossen et al., 2015b).

The seminal 1958 clinical report by Eiseman et al. documented successful FMT administration via fecal enema in four critically ill patients with pseudomembranous colitis, establishing the foundational protocol for modern microbiota-based therapies (Eiseman et al., 1958). Over subsequent decades, FMT has increasingly been implemented in clinical practice, demonstrating particular therapeutic efficacy in managing *Clostridium difficile* infection (CDI) and pseudomembranous colitis (Bowden et al., 1981; Russell et al., 2010; Tvede and Rask-Madsen, 1989; Yoon and Brandt, 2010). FMT administration modalities have expanded to include colonoscopic infusion (Persky and Brandt, 2000), transnasogastric tube delivery (Aas et al., 2003), transnasoduodenal tube infusion (van Nood et al., 2013), and supervised home-based protocols (Silverman et al., 2010), among others. Furthermore, emerging clinical evidence indicates FMT’s therapeutic potential for diarrhea (Gustafsson et al., 1998; Paterson et al., 1994), constipation, MetS (Vrieze et al., 2012), irritable bowel syndrome (IBS) (Andrews and Borody, 1993), and ulcerative colitis (UC) (Bennet and Brinkman, 1989; Borody et al., 2003), among other conditions. As research continues, FMT has emerged as a validated therapeutic modality for multiple gastrointestinal and systemic disorders.

**Figure 1:**
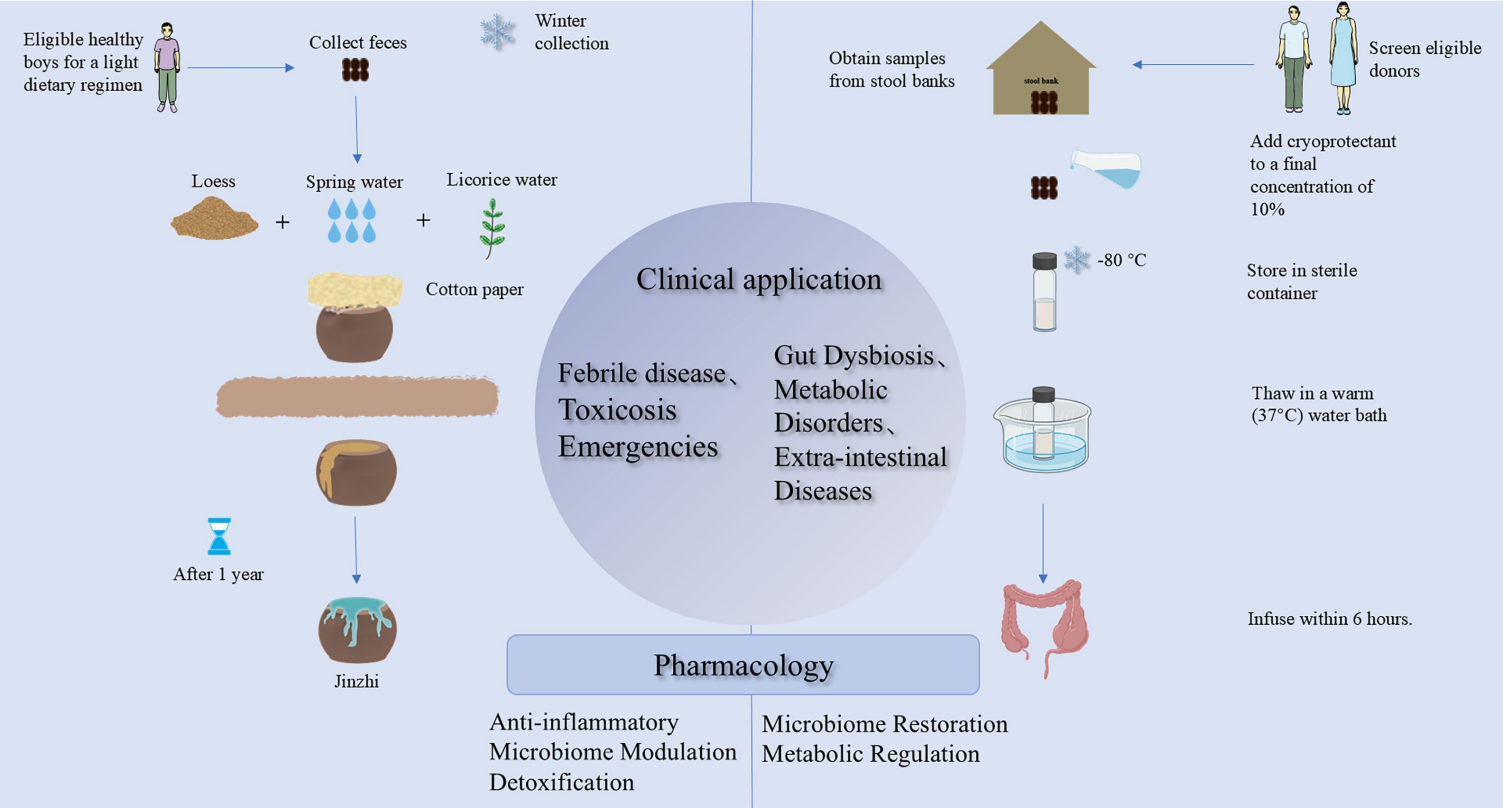
Comparison of the composition, preparation of Jinzhi and FMT’s suspensions, and their distinct therapeutic applications.

Although FMT was first formally documented in 1958, the conceptual origins of microbiotherapeutic interventions can be traced back to 2nd-century China (during the Eastern Han Dynasty). Early Chinese medical texts describe the therapeutic use of processed fecal preparations for toxin exposure management, with subsequent systematization into Jinzhi, a standardized formulation in traditional Chinese pharmacopeia (Figure 1). Throughout the historical evolution of fecal pharmacotherapy, the nomenclature of these preparations has undergone significant changes, concomitant with a continual expansion of their therapeutic indications. Similarly, Panchgavya therapy (or cow-derived product therapy) documented in Indian Ayurvedic medicine operates on principles comparable to microbiotic principles (Bajaj et al., 2022). Contemporary research has substantiated that its key component, cow dung, exhibits potent antibacterial and antifungal properties. Ayurvedic texts also describe the use of cow urine for treating various ailments, such as diarrhea, jaundice, and hemorrhoids; however, robust clinical evidence supporting these applications remains scarce. During World War II, historical accounts indicate that African Bedouins advised German soldiers stationed in North Africa to ingest fresh camel feces as a remedy for bacterial dysentery (LaMont, 2000). These historical precedents collectively illustrate that fecal-based therapies have been a geographically widespread and historically recurrent medical practice. As a modern embodiment of this ancient practice, FMT has demonstrated considerable therapeutic potential across multiple domains, particularly in gastrointestinal and metabolic diseases, since its contemporary inception. This review delineates the history of fecal therapy in Chinese medicine, focusing on the preparation protocol of its representative formulation, Jinzhi. By integrating contemporary scientific research, this article aims to preliminarily elucidate the underlying anti-inflammatory mechanisms, thereby proposing hypotheses to inform future research on optimizing donor screening and FMT preparation procedures.

## 2 Methods

### 2.1 Literature search strategy

A targeted literature search was performed to identify relevant publications up to March 2025. Electronic databases, including PubMed, Web of Science, and the China National Knowledge Infrastructure (CNKI), were queried. The search strategy combined key terms and their variants, such as: “Jinzhi”; “fecal microbiota transplantation” OR “FMT”; “donor”; “seasonal”; “administration”; “therap*” (to capture therapy, therapeutic, etc.); “flora characteristics” OR “microbial composition”; “loess”; and “prepar*” (to capture preparation, preparing, etc.). These terms were adapted for use in both English and Chinese databases.

### 2.2 Study selection criteria

Information regarding the historical practice of Jinzhi was primarily drawn from foundational texts of Traditional Chinese Medicine (TCM). For contemporary data on both Jinzhi and FMT, the search focused on studies detailing preparation methods, compositional analyses, clinical applications, and mechanistic research.

The selection of literature followed these criteria:

Inclusion: Classical TCM texts, peer-reviewed journal articles (in Chinese or English), and relevant reviews were included.

Exclusion: Publications for which the full text could not be accessed were excluded.

### 2.3 Evidence synthesis and analytical approach

Given the fundamental disparity in the sources, a distinct analytical approach was applied to historical and modern evidence:

#### 2.3.1 Analysis of historical evidence

Information about Jinzhi was extracted from classical TCM texts, including *Hua Tuo Shen Fang* (华佗神方, c. 145–208 AD) (Hua, 2011), *Jin Gui Yao Lue* (金匮要略, c. 150–219 AD) (Zhang, 2022), *Ben Cao Gang Mu* (本草纲目, 1578 AD) (Li, 2014), and *Wan Bing Hui Chun* (万病回春, 1587 AD). These texts were treated as primary historical records documenting traditional medical theory, empirical practices, and materia medica applications. The analysis focused on descriptively summarizing the documented preparation methods, purported indications, and theoretical rationales, without attempting to validate their clinical efficacy or biological mechanisms by modern standards.

#### 2.3.2 Analysis of modern evidence

Contemporary scientific literature on FMT and available experimental studies on Jinzhi were reviewed to establish the current state of knowledge. Priority was given to clinical trials, controlled experimental studies (*in vivo*/*in vitro*), and microbial compositional analyses published in peer-reviewed journals. The findings from modern research were summarized to outline established protocols, evidence of efficacy, and understood mechanisms.

#### 2.3.3 Comparative analytical framework

The core analytical method of this review was a structured comparative analysis. The descriptively summarized features of the historical Jinzhi protocol were systematically juxtaposed with the practices and findings of modern FMT across defined dimensions (e.g., donor selection, preparation, timing, administration). This juxtaposition was conducted to identify salient contrasts and convergences, with the explicit aim of generating novel, testable hypotheses for future scientific investigation (e.g., the potential influence of donor age, fermentation, or environmental microbiota). The review does not seek to equate historical practices with modern evidence but to use historical insights to inform future research questions in microbiome science.

### 2.4 Nature of this review

This is a narrative review. Its primary aim is not to provide a systematic aggregation of all existing evidence, but to construct a conceptual framework by comparing historical practices with contemporary science. We synthesize information from diverse sources, including classical TCM texts and modern experimental studies, to identify contrasts, generate novel hypotheses, and propose a forward-looking research agenda for microbiota-based therapeutics.

## 3 Origin and development of Jinzhi

The therapeutic application of processed human feces in TCM, systematically developed into the formulation known as Jinzhi, traces its conceptual origins to the Eastern Han Dynasty (25–220 A.D.). The earliest documented use appears in *Hua Tuo Shen Fang* (c. 145–208 AD), which describes the ingestion or topical application of filtered human fecal juice to treat wounds from venomous arrows. Shortly thereafter, *Jin Gui Yao Lue* (c. 150-219 AD) records its use for poisonous fungal poisoning. By the Eastern Jin Dynasty, Ge Hong’s *Emergency Formulas to Keep Close at Hand* (肘后备急方) (306-317 AD) (Ge, 2016) mentions a fermented preparation named Huanglong Tang, specifically noting that the product being “aged for a long time is good,” marking an early conceptual prototype of Jinzhi.

Over subsequent centuries, the preparation methods, therapeutic indications, and nomenclature of human feces-based drugs evolved: feces juice, human feces juice, feces clear, Huanglong Tang, Diqing (earth-filtered liquid), Huan Yuanshui, Jinzhi, and so on. By the Ming Dynasty (1368–1,644), the preparation method, specific usage, and the main treatment of diseases of the Jinzhi tended to be largely standardized.

### 3.1 Clarification between Jinzhi and Renzhonghuang

Notably, historical confusion persists between “Jinzhi” and “Renzhonghuang” (Human Middle Yellow) in classical medical texts. Although both are derived from human feces and share overlapping therapeutic claims, their preparation methods are distinct. Jinzhi is produced through the filtration and long-term underground fermentation of fresh feces. Conversely, Renzhonghuang is prepared by placing licorice root powder inside bamboo tubes, immersing them in a fecal pit during winter, and drying the contents in spring. The relative simplicity of Renzhonghuang’s preparation likely contributed to its continued, albeit limited, use, while the complex Jinzhi has become exceedingly rare.

### 3.2 Standardized preparation protocol

The preparation method for Jinzhi was comprehensively detailed in Ming Dynasty pharmacopeias, most notably in Li Shizhen’s *Ben Cao Gang Mu*, which refined earlier techniques such as the bamboo tube seepage method described in *Hua Tuo Shen Fang*. The standardized protocol, synthesized from classical texts, involves several critical stages:

1. Donor Selection: Rigorous criteria were emphasized, particularly in Gong Tingxian’s *Wan Bing Hui Chun* (1587 AD) (Gong, 2019). Optimal donors were healthy, prepubescent boys (typically aged 12–16 years), maintained on a bland vegetarian diet and abstaining from pungent foods (e.g., alliums) and meat. TCM theory posits that such individuals embody a “chunyang zhi ti (pure yang body)”, considered ideal for producing medicinal Jinzhi.
2. Timing and Collection: Ancient texts such as *Lei Gong Pao Zhi Yao Xing Jie* (雷剬炮制药性解, 1622 AD) (Li, 1998), *Ben Jing Feng yuan* (本经逢原, 1695 AD) (Zhang, 2007), and *Ben Cao Shu Gou Yuan* (本草述钩元, 1833 AD) (Yang, 1958) also emphasize that the Jinzhi should be prepared by choosing feces from the winter and twelfth lunar month (November–December of the Chinese lunar calendar).
3. Filtration Process: The collected feces were placed in a vessel, covered with cotton paper or cloth, and layered with loess (or red soil). Clean well water or mountain spring water was then used to repeatedly rinse and filter the mixture. The filtered liquid was collected in a new urn.
4. Long-term Fermentation: The sealed urn was buried underground for a minimum of 1 year, and often for decades. This prolonged anaerobic fermentation phase was considered essential, encapsulated in the adage “the older, the better.”
5. Final Product: Upon retrieval, the contents are stratified. The uppermost layer—a clear, yellowish, and odorless liquid—constituted the final Jinzhi product used for therapeutic purposes.

This historical protocol, characterized by strict donor selection, seasonal timing, loess filtration, and prolonged underground fermentation, establishes the foundational contrasts with modern FMT practices explored in this review.

## 4 Clinical indications of Jinzhi

Jinzhi, which exerts its medicinal effects through oral administration (Li and Zhuang, 2015; Xu et al., 2017), was widely used in ancient medical texts to “detoxify” and “relieve fever,” with therapeutic applications spanning acute conditions and infectious diseases (Table 1).

**Table 1:** Relevant descriptions of Jinzhi application in classical Chinese Medical texts.

Therapeutic category (TCM)	Specific application/Indication (Historical)	Key classical textual evidence
Detoxification (Jie Du)	Animal Venom and Insect Bites	*Hua Tuo Shen Fang*: For “poisonous stings of snakes and insects.”
	Mycotoxicosis (Mushroom Poisoning)	*Jin Gui Yao Lue*: For “mycotoxicosis manifesting mental confusion.”
	Plant Poisoning	*Bei Ji Qian Jin Yao Fang* (c. 652 AD) (Sun, 1982), *Ben Cao Gang Mu*: For toxicity from wild kudzu/taro.
	Drug Overdose	*Qian Zhai Jian Xiao Fang* (Wang, 1853): For dizziness/mania from opium.
Heat-Clearing (Qing Re)	Febrile Delirium	*Ben Jing Feng Yuan*: For “Wen Re presenting with Shi Xing Hun Re.”
	Eruptive Epidemic Diseases	*Chong Ding Guang Wen Re Lun* (1911 AD) (He, 1960): For “warm toxin, mumps, macula, scarlatina.”
	Localized Severe Infections	*Zeng Ding Tong Su Shang Han Lun* (1932 AD) (He, 2004): For “erysipelas facialis, red diaphragm typhoid fever.”
	Syndromic Use in “Warm Diseases”	*Wen Re Feng Yuan* (1900 AD) (Liu, 1959): For latent pathogens erupting as macules/rash.

## 5 Modern mechanistic research on Jinzhi

Modern pharmacological and mechanistic studies of Jinzhi have proven highly challenging, primarily due to ethical constraints and its gradual disappearance from clinical practice. Our literature search on Jinzhi identified several studies focusing on its effects on inflammatory responses in murine models. For instance, a controlled trial demonstrated that Jinzhi exerts a therapeutic effect in septic mice by regulating the release of pro-inflammatory factors, including IL-1β, IL-6, and TNF-*α*, thereby controlling inflammatory progression. However, the precise mechanism underlying this effect remains unexplored (Tong et al., 2023). A subsequent, more in-depth study revealed that Jinzhi improves the intestinal mucosal barrier in sepsis. Comparative experiments indicated that this protection is achieved through the regulation of LC3I, LC3II, and Caspase-3 expression, which modulates cellular autophagy and apoptosis, thus effectively preserving the intestinal mucosal barrier and mitigating the septic inflammatory response (Ma and Xu, 2024). Although current research on Jinzhi remains limited, animal studies indicate that it may modulate systemic inflammation. The gut microbial community within Jinzhi undergoes substantial and complex alterations during its multi-year underground fermentation, which renders the interspecies interactions in this community difficult to predict. Thus, further in-depth investigations are warranted to validate these preliminary findings and elucidate the underlying regulatory mechanisms.

Notably, Jinzhi, a characteristic bitter-cold herb in TCM used to treat heat-related illnesses, has long been considered to correspond to the modern disease model of infection, fever, and inflammation. This traditional concept is consistent with recent FMT findings, which demonstrate efficacy in ameliorating inflammatory responses. Critically, they share a fundamental characteristic: they are processed derivatives of human feces. However, the preparation of Jinzhi is considerably more complex, involving a decades-long fermentation process.

## 6 Differences between Jinzhi and FMT

### 6.1 Difference in preparation time

Jinzhi is primarily prepared during the winter months, whereas FMT demonstrates no distinct seasonal dependency.

Jinzhi is typically prepared during the month surrounding the winter solstice for two principal reasons: First, the underdevelopment of refrigeration technology in ancient times necessitated winter preparation to minimize specimen degradation and inhibit microbial proliferation and decomposition; Second, simplified winter diets facilitated donor selection due to reduced dietary variability. Furthermore, the holistic theory of Traditional Chinese Medicine (TCM) posits that seasonal changes influence the composition of human gut microbiota. Previous research has revealed higher gut microbial diversity in winter than in summer, with seasonal variations in *Actinobacteria, Bacteroidetes*, and the *Firmicutes/Bacteroidetes* ratio (F/B ratio) (Davenport et al., 2014; Koliada et al., 2020). Such seasonal fluctuations may contribute to the development of obesity, a known risk factor for IBD (Di Pierro, 2021; Stojanov et al., 2020). Subsequent investigations have identified low-temperature fermentation as a crucial step in Jinzhi preparation. This process significantly increases probiotic abundance and Short-Chain Fatty Acids (SCFAs) production while exhibiting anti-inflammatory properties (Fu, 2020). This validates the ancient adage that “aged for a long time is good” and provides scientific support for the practice of long-term Jinzhi fermentation. Therefore, the traditional practice for Jinzhi preparation is hypothesized to have potentially enhanced bacterial community diversity while possibly mitigating enteric disease risks. Our literature review revealed that current FMT protocols rarely address the timing of stool sample collection, an oversight that warrants attention. Given that seasonal variations in the human gut microbiota are largely driven by dietary patterns (Koliada et al., 2020), incorporating seasonal considerations into sample collection protocols is crucial. It will undoubtedly inform the design of future FMT-related research.

### 6.2 Differences in donor sources

**Figure 2:**
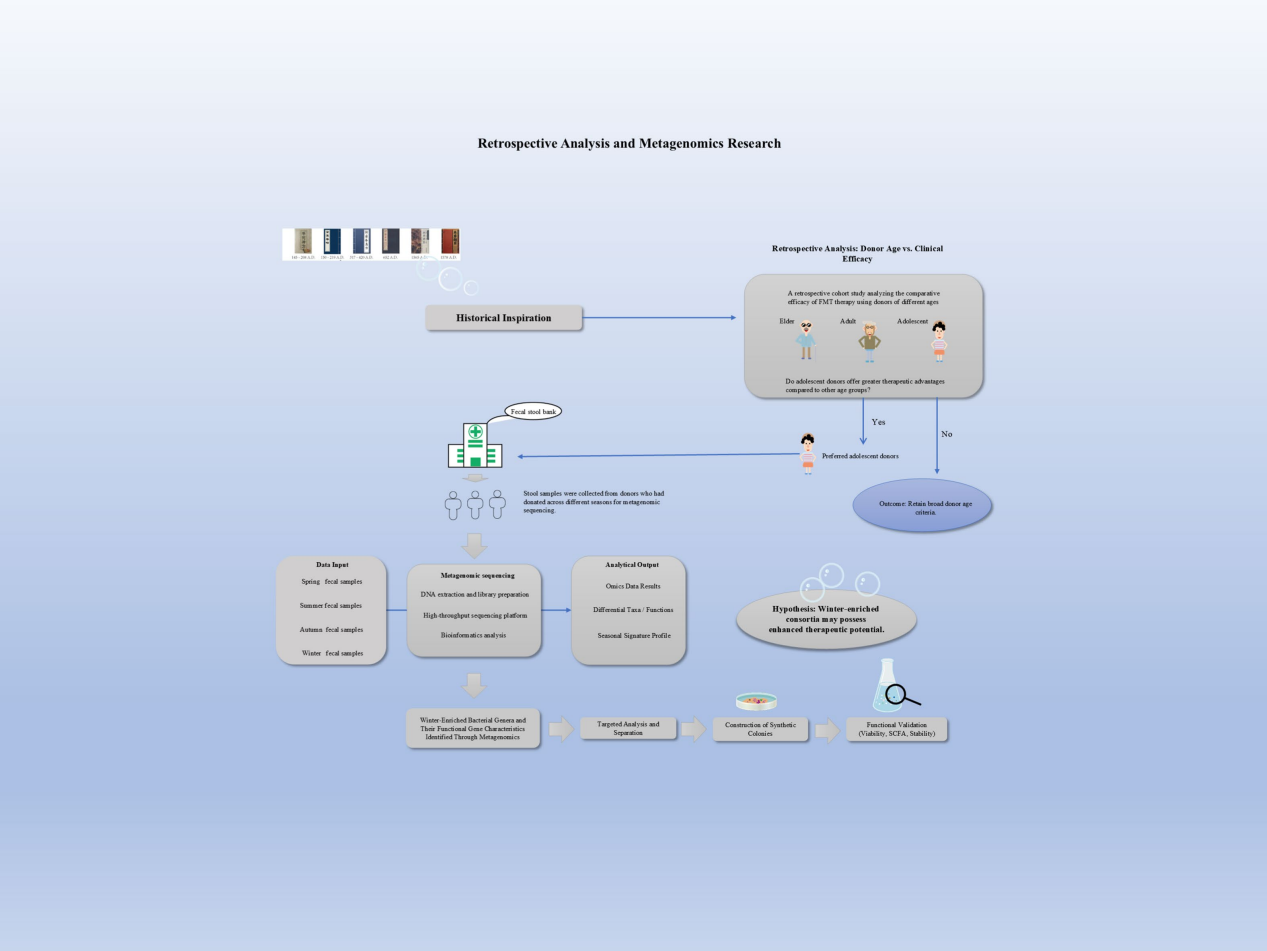
Workflow for optimizing FMT. Initial assessment of adolescent donor efficacy via retrospective analysis. Subsequent steps include cross-seasonal fecal sampling from selected donors, metagenomic sequencing to identify beneficial microbiota, and construction of synthetic consortia for experimental validation.

Jinzhi exclusively utilizes fecal material from healthy boys aged 11 to 12 years. Conversely, as per international consensus, modern donor selection for FMT encompasses a broader demographic range. Donor age criteria typically fall under 50 or 60 years, with some guidelines considering individuals under 30 suitable donors (Zhang et al., 2022). The gut microbiomes of children and adults exhibit striking differences across developmental stages. A study simulating Jinzhi preparation conditions found that the intestines of healthy boys aged 12–14 years were rich in potential probiotics, including *Faecalibacterium, Blautia, Bifidobacterium*, and *Roseburia*, which collectively constituted 45.09% of the total microbiota (Fu, 2020). Notably, conditionally pathogenic genera such as Escherichia/Shigella, Enterobacter, and Klebsiella, which were commonly detected in adults, were nearly undetectable in the feces of these boys. The gut microbiota undergoes significant changes throughout childhood, gradually stabilizing from infancy to later developmental stages (Stewart et al., 2018). While pediatric and adult microbiota share core phyla like Bacteroidetes and Firmicutes, the abundance of Bacteroidetes is often lower in children. 16SrDNA sequencing has revealed that although phylogenetically similar, they differ compositionally, with significant differences at the genus level and marked pediatric-adult divergences in microbial abundance, diversity, and community structure (Karmisholt Grosen et al., 2025). A recent prospective cohort study has specifically examined the impact of donor characteristics on FMT outcomes for rCDI. Multivariate models confirmed that donor age stratification was an independent factor influencing FMT outcomes, with rCDI remission rates gradually decreasing across donor age groups from 24 to 65 years (Grosen et al., 2025). However, no relevant studies were retrieved in which donor ages spanned from 12 to 50 years. Therefore, the use of adolescent donors is a specific historical practice. Its potential to confer additional therapeutic value in a modern context remains a hypothesis requiring rigorous validation (Figure 2).

The comparative efficacy of single-donor versus multi-donor FMT remains understudied, with current evidence showing considerable heterogeneity. While multi-donor fecal samples are associated with higher microbial diversity (Paramsothy et al., 2017), clinical outcomes vary. For instance, a Phase II RCT in cirrhotic patients reported a significantly lower recurrence rate with dual-donor FMT (9%) compared to placebo (40%), demonstrating enhanced efficacy compared with earlier findings (Shawcross and Patel, 2025). Conversely, a multicenter double-blind randomized trial for active ulcerative colitis found no significant difference in clinical remission rates between allogeneic multi-donor and autologous FMT (Caenepeel et al., 2025). Consequently, the comparative efficacy of multi-donor versus single-donor FMT warrants further investigation.

### 6.3 Differences in preparation methods

#### 6.3.1 Preparation of FMT

In 2025, Gong et al. proposed preparation standards for laboratory fecal suspensions, providing comprehensive details on sample collection, storage, suspension buffer selection, homogenization, filtration, centrifugation, and cryopreservation (Gong et al., 2025). This work has made a significant contribution to the standardization of FMT. However, a review of current international guidelines from the United States, Europe, China, and other regions reveals that therapeutic FMT protocols still primarily focus on donor selection criteria to exclude infectious diseases and conditions that may disrupt the gut microbiota (Burz et al., 2019; Cammarota et al., 2017, 2019; Society of Parenteral and Enteral Nutrition, Chinese Medical Association, & Microecology Professional Committee of Shanghai Preventive Medicine Association, 2022; Kump et al., 2014; Rossier et al., 2023; Lopetuso et al., 2023; Gweon et al., 2022). While a growing body of research is beginning to recognize the profound influence of donors on the microbial communities transferred during FMT, standardizing these processes and personalizing FMT for individual recipients remain pressing and unresolved challenges.

#### 6.3.2 Preparation of Jinzhi

1. In preparation protocols, Jinzhi predominantly employs mountain spring water or well water for filtration, contrasting with FMT’s standardized use of sterile saline solution.

Studies analyzing different types of drinking water contamination and microbial community characteristics revealed that well water exhibits: (1) excessive total colony counts exceeding safety thresholds; (2) remarkably high microbial diversity indices; (3) significantly elevated detection rates of pathogenic species (Cui et al., 2023). Contrarily, murine models indicate that hydrological parameters (consumption volume) rather than mineral composition predominantly modulate gut microbiota profiles (Kim and Shin, 2023). The use of mountain spring or well water in Jinzhi filtration likely introduces environmental microbiota—a factor absent in modern FMT’s sterile saline preparations. The functional consequences of this difference are unknown. Moreover, how subterranean burial affects the microbial community and the precise role of these water sources themselves require further investigation.

2. The Jinzhi preparation process incorporates stratified filtration through loess (laterite) layers.

Soil exposure was found to enhance intestinal anti-inflammatory activity in mice, upregulating expression of anti-inflammatory factors (Foxp3, CTLA4) and the anti-inflammatory cytokine IL-10, and to affect gut microbiota structure, reducing the Firmicutes/Bacteroidetes (F/B ratio). *Bacteroidetes* play significant roles in intestinal metabolism, such as the capsular polysaccharide of *B. fragilis NCTC9343* and sphingomyelins, which demonstrate therapeutic potential in multifactorial colitis models (Fernandez-Julia et al., 2021; Hooper et al., 2002; Tan et al., 2019; Zhou et al., 2024). Furthermore, Liddicoat et al. (2018) demonstrated that soil-derived microbiota diversity positively correlates with host health outcomes. Research on soil microbiota exposure suggests a potential mechanistic basis for this historical practice; the loess filtration process could have inadvertently introduced microbes or minerals that influence anti-inflammatory pathways and promote intestinal metabolism.

3. Historical records indicate that licorice (*Glycyrrhiza glabra*) water is also added to the Jinzhi preparation.

As a traditional Chinese medicine, licorice has heat-clearing and detoxifying effects that complement Jinzhi in the treatment of febrile illnesses. Studies have shown that the various components of licorice (saponins, flavonoids, etc.) have distinct pharmacological effects. For instance, *β*-glycine, the main metabolite of glycyrrhizic acid (a saponin compound), has anti-inflammatory activity and can inhibit the self-replication of a variety of viruses; specific flavonoids have been identified as active constituents of licorice against methicillin-resistant *Staphylococcus aureus* (MRSA), and have been demonstrated to restore the effect of benzoxicillin and β-lactam antibiotics against methicillin-resistant *S. aureus*. Furthermore, glycyrrhizic acid has antiulcer properties, leading to ulcer healing by promoting mucus secretion and cell proliferation in the stomach; isoglycoside and glycyrrhizin show gastroprotective activity by reducing inflammation, stimulating cellular antioxidants, and regulating gastric acid secretion (Asl and Hosseinzadeh, 2008; Hosseinzadeh and Nassiri-Asl, 2015). From a TCM perspective, the addition of licorice water also modulates the medicinal properties of the formulation.

### 6.4 Differences in storage methods

Regarding storage, the Jinzhi preparation is sealed post-filtration and stored subterraneously for over 1 year before application, during which the microbial dynamics of prolonged fermentation remain uncharacterized. Conversely, FMT protocols require immediate cryopreservation at −80 °C using cryoprotectant agents upon donor specimen collection, followed by controlled ambient-temperature thawing before therapeutic infusion.

### 6.5 Differences in the mode of administration

Jinzhi is typically administered orally, either as monotherapy or combined with TCM formulations. For example, in the *Chong Ding Guang Wen Re Lun*, it is recorded that “modified Zhuye Shigao Tang” is taken with Jinzhi added to a series of Chinese medicines.

There are several routes of FMT administration, including oral capsules, nasogastric/nasoduodenal tubes (NGT/NDT), enemas, or colonoscopies, and the optimal route remains inconclusive. Retrospective studies and evaluations have shown that FMT capsules and enemas have different remission rates in patients with rCDI, but no significant difference in recurrence rates; colonoscopic administration has the highest cure rate, followed by capsules, enemas, and NGT. Meanwhile, Randomized controlled trials confirm colonoscopic FMT’s efficacy advantage over nasogastric delivery, with an overall treatment efficacy of 100%, while noting procedure-specific risks, including endoscopic perforation and aspiration pneumonia (Fadda, 2020; Ramai et al., 2021; Svensson et al., 2022). Subsequent studies investigating cure rates across different routes of administration for rCDI (Vaughn et al., 2023) report comparable cure rates across administration routes, with colonoscopic and capsule methods demonstrating relative advantages over enema and nasogastric approaches. Conversely, licorice components in Jinzhi preparation may alleviate gastrointestinal adverse effects, including emesis and reflux.

### 6.6 Differences in the types of diseases treated

Jinzhi demonstrates broader therapeutic applications than FMT, particularly in its traditional use for “detoxifying all poisons and healing all sores (neutralizing toxins and resolving suppurative lesions).” (Table 2).

**Table 2:** Comparative therapeutic profiles of Jinzhi and fecal microbiota transplantation.

**Dimension**	**Jinzhi**	**FMT**
Primary indications	Wen Bing (warm-pathogen disease) Warm-heat disease Shu Wen (summerheat-warmth disease) Macula Shi Wen (dampness-warmth) Wen Yi (epidemic febrile diseases) Lanhou Sha (scarlatina) Smallpox Plague Opium poisoning Sepsis	Recurrent Clostridioides difficile infection (rCDI) Inflammatory bowel disease (IBD) Irritable bowel syndrome (IBS) Metabolic syndrome (MetS) Hepatic encephalopathy (HE) Chronic obstructive pulmonary disease (COPD) Hepatocellular carcinoma (HCC)
Evidence basis	Historical records^a^: *Ben Cao Gang Mu* *Wen Re Feng Yuan* *Chong Ding Guang Wen Re Lun* *and other documented sources* Microbial composition profiling via 16SrRNA sequencing (Xu et al., 2020)	RCTs^b^ (Costello et al., 2019; Moayyedi et al., 2015; Rossen et al., 2015a; Paramsothy et al., 2017) Meta-analyses^b^ (Wang et al., n.d.)
Evidence level	Level IV: Historical empirical records	Level I-II: Controlled clinical studies

^a^Representative classical texts shown (see Figure 1 for complete sources). ^b^FMT data from 2015 to 2025 clinical trials and meta-analysis.

The therapeutic potential of Jinzhi was systematically developed during the Ming-Qing period, particularly through the Warm Disease School (*Wen Bing Xue Pai*). JinZhi is primarily indicated for critical febrile diseases in TCM, with documented efficacy in “neutralizing all toxins and healing all sores” (解一切毒, 疗一切疮). This preparation exhibits a salty-mildly bitter flavor with a cold nature, targeting heat-syndrome disorders, including warm-pathogen disease, warm-heat syndrome, and summerheat-warmth disease. Classical TCM pharmacopeia records its use against epidemic diseases such as macula, scarlatina, and plague, particularly for critically ill warm-disease patients with high-grade fever and polydipsia, delirium with unconsciousness, and acute pharyngodynia. Notably, some have proposed that TCM’s concept of exogenous febrile diseases aligns with modern infectious disease studies examining the infection-inflammation-fever pathway. This suggests a certain correlation between the “heat syndrome” in TCM and inflammation in modern medicine, an observation tentatively supported by studies showing Jinzhi can regulate inflammatory factors in septic mouse models.

Building upon its established efficacy for rCDI, the therapeutic landscape of FMT has rapidly expanded to encompass a spectrum of other conditions.

In gastrointestinal diseases, FMT has shown promising outcomes for a spectrum of disorders, including IBD, IBS and Crohn’s disease (CD) (Sokol et al., 2020). For ulcerative colitis (UC), a meta-analysis of five randomized controlled trials (RCTs) demonstrated superior clinical remission rates in FMT recipients compared to placebo (Costello et al., 2019; Haifer et al., 2022; Moayyedi et al., 2015; Paramsothy et al., 2017; Rossen et al., 2015a).

Beyond the gut, FMT is being explored for its systemic effects. In MetS, a large-scale study (Wang et al., n.d.) observed that allogeneic FMT significantly improved lipid profiles, elevating high-density lipoprotein cholesterol (HDL-C) and reducing low-density lipoprotein cholesterol (LDL-C) at 12-week follow-up. Preliminary evidence also suggests potential applications in hepatic encephalopathy (HE) and Chronic obstructive pulmonary disease (COPD), highlighting its role in modulating the gut-liver and gut-lung axes, respectively (Budden et al., 2024; Shawcross and Patel, 2025).

The potential of FMT extends even to oncology, as illustrated by a mechanistic study on hepatocellular carcinoma (HCC) metastasis (Deng et al., 2025). This research identified that FMT from healthy donors could suppress neutrophil-induced inflammation by inhibiting the NETs-CD31 axis, thereby countering the pro-metastatic effects of a dysbiotic microbiota and inhibiting tumor progression in model mice (Cristinziano et al., 2022; Herre et al., 2023; Papayannopoulos, 2018).

Despite this promise, the clinical translation of FMT faces significant standardization hurdles, including unstandardized preparation protocols, undetermined optimal dosage, and a lack of consensus on the most effective administration route (Bethlehem et al., 2024). Recent large-scale studies have provided critical insights for optimizing FMT protocols. A Danish real-world cohort study (*n* = 1,170) confirmed that repeated FMT administration yields a significantly higher cure rate for CDI than a single dose (81% vs. 60%) (Paaske et al., 2025). This study also reported comparable efficacy for oral capsules and colonoscopy-delivered FMT, and identified that a longer duration of antibiotic pretreatment was associated with improved cure rates, a finding corroborated by other research (Groenewegen et al., 2025).

### 6.7 Differences in flora characteristics

Xu Jianfeng’s team collected Jinzhi samples from Ciqi Palace in Huaqiao, Quanzhou. It used 16SrDNA sequencing to compare their bacterial composition with that of FMT samples and identified 10 bacterial groups with significant abundance variations (as shown in Table 3). Animal experiments further showed that Jinzhi significantly regulated the intestinal microecology in lipopolysaccharide (LPS)-induced septic mice, reversing the decreased Firmicutes/Bacteroidetes ratio and increasing inflammation and intestinal mucosal damage, and promoting intestinal health, which provided a microbiological basis for the therapeutic efficacy of Jinzhi (Luan et al., 2024; Xu et al., 2020; Xu et al., 2019). Analyses reveal a marked enrichment of *Proteobacteria* in Jinzhi’s microbial community (Section 6.7, Table 2), a phylum frequently linked to gut dysbiosis and encompassing numerous opportunistic pathogens. This taxonomic profile appears paradoxical given the preparation’s demonstrated anti-inflammatory properties in septic mouse models (Section 4, Modern Mechanistic Research on Jinzhi). This discrepancy suggests that the observed therapeutic effects may not be attributable to individual bacterial taxa but rather emerge from the integrated functionality of the entire, long-term fermented ecosystem. The unique anaerobic fermentation conditions may select for microbial strains with specific functional attributes or drive the production of beneficial metabolites, thereby overriding conventional risk assessments based solely on taxonomy.

**Table 3:** Comparison of dominant bacterial communities at different levels between Jinzhi and suspension.

	Jinzhi	Fecal suspension
Phylum	*Proteobacteria*	*Firmicutes* *Bacteroidetes* *Fusobacteria*
Class	*Gammaproteobacteria* *Alphaproteobacteria* *Betaproteobacteria*	*Bacteroidia* *Clostridi*
Order	*Pseudomonadales* *Burkholderiales* *Rhizobiales* *Others*	*Bacteroidales* *Clostridiales* *Bifidobacteriales*
Family	*Pseudomonadaceae* *Comamonadaceae* *Rhizobiaceae* *others*	*Prevotellaceae* *Bacteroidaceae* *Ruminococcaceae* *Fusobacteriaceae* *Lachnospiraceae*
Genus	*Pseudomonas* *Hydrogenophaga* *Aeromonas* *unidentified_Gammaproteobacteria*	*Prevotella_9* *Bacteroides* *Faecalibactgerium [Eubacterium]_coprostanoligenes_group* *Megamonas*
Species	*Pseudomonas_mendocina* *Pseudomonas_Luteola* *Hydrogenophaga_intermedia* *Gamma_proteobacterium_HdN1*	*Bacteroides_coprocola* *Bacteroides_plebeius* *Bacteroides_coprophilus* *Bacteroides_Uniformis* *Fusobacterium_Mortiferum* *Bifidobacterium_adolescentis*

To harness the potential insights from Jinzhi while addressing the safety concerns inherent in its traditional preparation, a structured translational research framework is proposed:

1. Functional Deconstruction: Utilize multi-omics technologies (metagenomics, metabolomics) to systematically identify the key bioactive metabolites and functional genetic pathways that underpin Jinzhi’s observed effects in model systems.
2. Synthetic Reconstruction: Based on these findings, employ principles of synthetic microbial ecology to construct defined consortia composed of well-characterized and clinically vetted bacterial strains. The goal is to recapitulate the putative beneficial functions within a controlled, reproducible, and safer product, thereby decoupling the hypothesized therapeutic principles from the risks associated with the raw, fermented source material.

## 7 Safety, ethical, and regulatory considerations

### 7.1 Biosafety and standardization challenges

The traditional preparation of Jinzhi involves prolonged, uncontrolled subterranean fermentation. This process results in a final product with a complex, poorly characterized microbial composition, presenting inherent biosafety risks absent in modern, regulated FMT protocols. These risks include the potential enrichment of opportunistic pathogens, undiscovered viral agents, or environmental toxins during the extended fermentation period. Our comparative analysis (Section 5.7) confirms that Jinzhi harbors a microbiota distinct from contemporary FMT preparations, including a notable enrichment of Proteobacteria, a phylum often associated with dysbiosis. Therefore, directly translating the historical Jinzhi protocol into clinical practice is not feasible under modern regulatory frameworks, which mandate rigorous donor screening, controlled manufacturing environments, and well-defined final products. The future value of Jinzhi lies not in its direct replication, but in using its principles, such as the potential functional benefits of long-term microbial succession, to inspire the *de novo* design of safer, standardized, synthetic microbial consortia or defined metabolite preparations.

### 7.2 Contemporary ethical and practical dilemmas

The historical protocol’s exclusive reliance on adolescent donors (as documented in Wan Bing Hui Chun) poses significant and insurmountable ethical challenges for modern translation. First, the classical texts provide insufficient detail regarding the duration and specifics of the prescribed vegetarian diet, making the protocol scientifically irreproducible. More critically, there is no contemporary clinical evidence to substantiate the superior therapeutic efficacy of microbiota derived from adolescent donors in FMT. Imposing a long-term, restrictive diet on healthy minors for the purpose of donor material collection would expose them to unknown risks regarding nutritional adequacy and development. This contravenes the core ethical principles of beneficence and non-maleficence, as well as the requirement for informed consent from a vulnerable population. Consequently, the donor selection criterion of Jinzhi cannot be adopted in contemporary practice. Its conceptual value is instead in prompting investigation into whether specific functional attributes of a “healthier” or more resilient microbiome, potentially more common in youth, can be identified and then replicated through alternative, ethical means.

## 8 Discussion

This review has systematically compared the historical TCM preparation Jinzhi with modern FMT, highlighting key methodological distinctions. Rather than presenting definitive solutions, this analysis aims to translate the empirical practices of Jinzhi into a series of compelling, testable hypotheses that could inform the future of microbiota-based therapeutics.

We hypothesize that the distinctive aspects of the Jinzhi protocol, commonly set aside as historical practice, could serve as a source of inspiration. The specific questions they raise present underexplored avenues for future hypothesis-driven FMT development. Specifically, we hypothesize that: (1) The selective use of adolescent donors, by leveraging a potentially more robust and beneficial gut microbiota, could enhance the microbial quality of transplant material; (2) The loess filtration and long-term fermentation processes were not merely preservation techniques but may have functionally shaped the microbiota, potentially enriching for anti-inflammatory metabolites and fostering a stable, synergistic microbial consortium through *ex vivo* ecological succession.

These historical practices contrast with the immediate use of frozen material in contemporary FMT, which may overlook the potential benefits of microbial maturation and environmental interaction. The challenge and opportunity now lie in applying modern scientific rigor to validate these ancient concepts.

## 9 Conclusion

Therefore, we suggest a forward-looking research agenda to experimentally test these premises. Key priorities include utilizing multi-omics to decipher the microbial and metabolic dynamics of long-term fermentation, and conducting controlled studies to evaluate the functional impact of young donor microbiota and soil-derived microbes on therapeutic outcomes.

**Figure 3:**
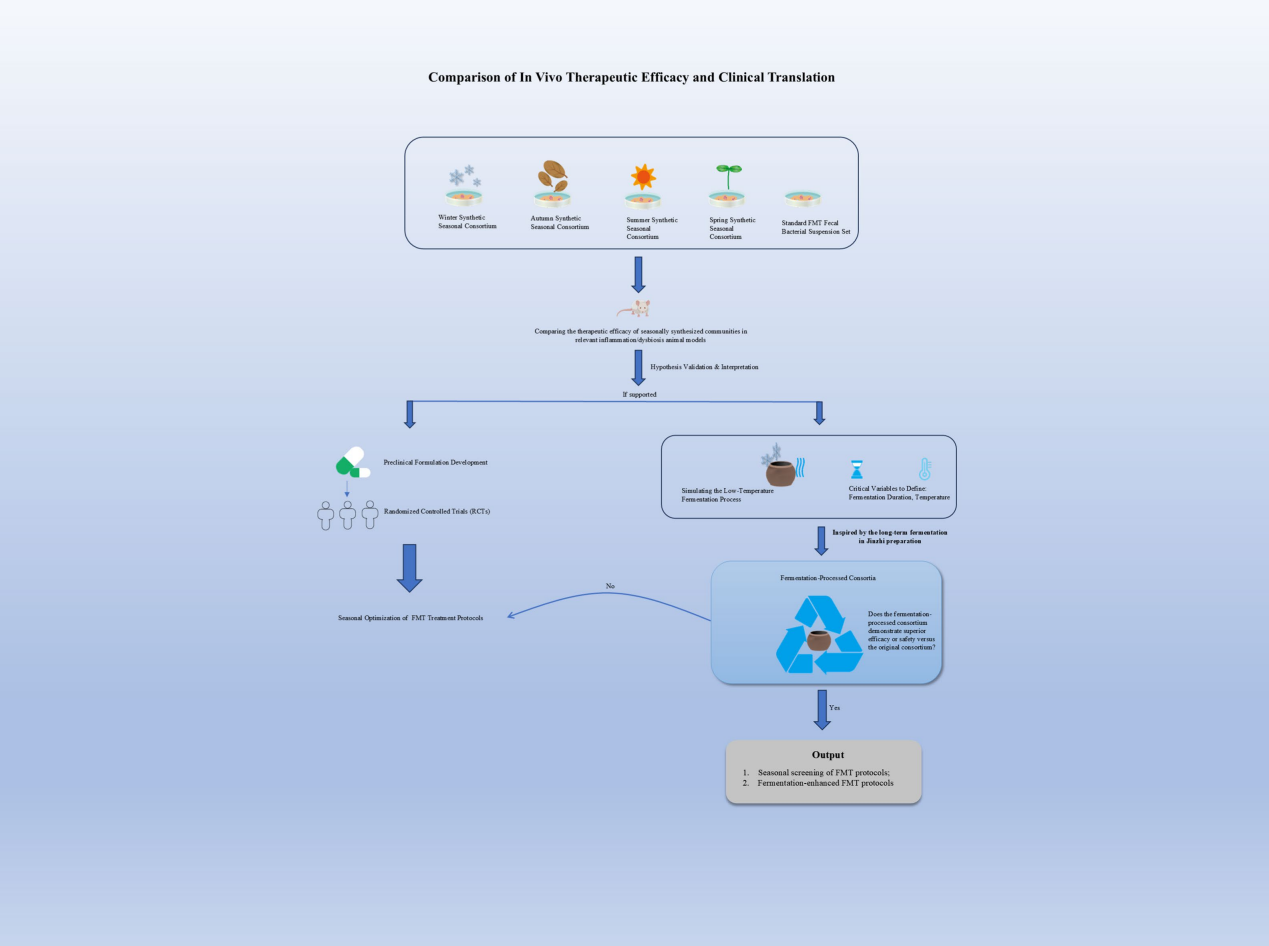
Comparative experiments evaluate the therapeutic advantage of winter gut microbiota, with subsequent expansion to RCTs for clinical validation. Parallel studies assess the impact of low-temperature fermentation on efficacy, guiding future translational development.

In summary, this review posits that Jinzhi’s preparation methodology offers a valuable conceptual framework, rather than a direct recipe, for innovating FMT. By rigorously investigating the hypotheses of donor selection, environmental augmentation, and controlled fermentation, the FMT field may discover novel strategies to move beyond simple transplantation towards the engineered cultivation of more effective and reliable microbiome therapeutics. The integration of this historical wisdom with cutting-edge science holds the potential to open new, promising frontiers in the field (Figure 3).

## Glossary

FMT: fecal microbiota transplantation
IBD: inflammatory bowel disease
CDI: *Clostridium difficile* infection
SCFAs: Short-Chain Fatty Acids
NGT: nasogastric tubes
COPD: Chronic obstructive pulmonary disease
MetS: metabolic syndrome
LDL-C: low-density lipoprotein cholesterol
HE: hepatic encephalopathy
TCM: Traditional Chinese Medicine
RCT: Randomized Controlled Trial
rCDI: recurrent *Clostridioides difficile* infection
UC: Ulcerative colitis
IBS: irritable bowel syndrome
NDT: nasoduodenal tubes
LPS: lipopolysaccharide
F/B ratio: Firmicutes/Bacteroidetes ratio
HDL-C: high-density lipoprotein cholesterol
CD: Crohn’s disease
MRSA: methicillin-resistant *Staphylococcus*
HCC: Hepatocellular carcinoma
